# Reliability demonstration test for load-sharing systems with exponential and Weibull components

**DOI:** 10.1371/journal.pone.0189863

**Published:** 2017-12-28

**Authors:** Jianyu Xu, Qingpei Hu, Dan Yu, Min Xie

**Affiliations:** 1 City University of Hong Kong Shenzhen Research Institute, Shenzhen, China; 2 Academy of Mathematics and Systems Science, Chinese Academy of Sciences, Beijing, China; 3 City University of Hong Kong, Hong Kong SAR, China; Universidad de Valladolid, SPAIN

## Abstract

Conducting a Reliability Demonstration Test (RDT) is a crucial step in production. Products are tested under certain schemes to demonstrate whether their reliability indices reach pre-specified thresholds. Test schemes for RDT have been studied in different situations, e.g., lifetime testing, degradation testing and accelerated testing. Systems designed with several structures are also investigated in many RDT plans. Despite the availability of a range of test plans for different systems, RDT planning for load-sharing systems hasn’t yet received the attention it deserves. In this paper, we propose a demonstration method for two specific types of load-sharing systems with components subject to two distributions: exponential and Weibull. Based on the assumptions and interpretations made in several previous works on such load-sharing systems, we set the mean time to failure (MTTF) of the total system as the demonstration target. We represent the MTTF as a summation of mean time between successive component failures. Next, we introduce generalized test statistics for both the underlying distributions. Finally, RDT plans for the two types of systems are established on the basis of these test statistics.

## Introduction

Reliability demonstration tests (RDT) aim to verify whether the reliability target for a certain system has reached a pre-specified threshold. They are essential in the industry before products are allowed to be marketed. Conventionally, RDT comprises a series of tests on product lifetime or degradation performance. During the tests, failures and degradation are examined according to the type of tests. A test on target reliability measurement can be summarized as a statistical hypothesis test with certain restrictions pertaining to risks and costs. RDT plans are established on the basis of the results of such tests. Popular quality standards such as MIL-HDBK-781A (1987) and IEC-1123 (1991) contain detailed test schemes based on lifetime data under different conditions. There is also a significant volume of literature on the establishment of demonstration tests under various conditions, e.g., [1], [2].

Test plans for demonstration investigated in related works can be classified into several categories according to their test schemes, lifetime distributions, degradation models and other experimental conditions. According to [3–5], traditional RDT demonstration tests are based on lifetime data that can be further divided into two types. Test plans based on failure numbers have been studied by scholars such as [6], [7]. In such test schemes, only binary data are considered and the test plan is established on the basis of a binomial distribution. Test plans based on failure times are also investigated by many scholars, e.g., [8], [9]. In such a test scheme, specific distribution assumptions are made to incorporate failure time information. In lifetime demonstration tests, a variety of test conditions (such as accelerated life tests), different situations (such as zero failure scenarios) and different system structures (such as k-out-of-n) are introduced. Prior information can be incorporated in such tests while designing the test plans, e.g., [9], [10]. Further, considering the long life span for some recent products, RDTs based on degradation have been developed for such situations, e.g., [11], [12]. Test plans incorporating both lifetime data and degradation data simultaneously have also been investigated, e.g., [13].

As a result of the high structural complexity found in recent systems, RDT for systems based on component-level experimental information is becoming increasingly difficult. The need for specific demonstration test plans executed on various systems is causing increasing concern. Although they have been studied for years, systems with dependent components have been receiving increasing attention due to the general existence of dependencies among components or subsystems. An important system modeling such dependencies is the load-sharing system. Consider a system consisting of a number of components with each failing one by one. After one component fails, the total load on the system is redistributed among the remaining components. This is referred to as *load-sharing, [14–17]*. Several applications of load-sharing systems have been investigated from the viewpoints of reliability modeling and assessment, e.g., for ([18]), power grids ([19]) and gear systems ([20]). Generalized model for load-sharing systems are studied in [21–23]. Though load-sharing plays an important role in system reliability modeling, maintenance and optimization design, few specific demonstration test plans have been established for such systems. Meanwhile, most of the existing demonstration methods reveal great difficulties in the application of *load-sharing*. The reason might be the fact that in a *load-sharing* system, due to the successive failure of each component, a series of failure times is constantly observed prior to the final failure of the total system. Since different stages between components’ failures do not share the same set of model parameters, this may act as a barrier while building the proper test plan for the RDT problem. Further, system-level tests are particularly time-consuming because load-sharing systems are generally designed for a high reliability. In this paper, we propose a reliability demonstration method for *load-sharing* systems based on the successive failure times of components. Though a common target of an RDT problem is system reliability at a certain mission time, system MTTF is also an important reliability index especially for systems designed with high reliability levels such as load-sharing systems and repairable systems [24]. We choose system MTTF as the demonstration target in our work. We constrain the underlying lifetime distribution of each component to be either an exponential or a Weibull distribution. Based on the previous literature about parameter estimation of load-sharing systems, we deduce the MTTF of the whole system. However, as we will show in the following sections, no ordinary test statistics can be constructed to execute a hypothesis test plan on the RDT problem in (1) for such a system. To solve the problem, we introduce the approach of generalized p-value test which was proposed first in Tsui and Weerahandi [25] which is recently extended and applied in [26] and [27]. Next, we will describe the implementation of this approach used to solve the RDT problem in detail. Finally, a test plan is proposed through the establishment of a specific generalized test statistic.

The rest of this paper is organized as follows. Section 2 gives a brief formulation of RDT and generalized test method. Section 3 reviews load-sharing systems with exponential and Weibull underlying lifetime distribution and MTTF under both situations are deduced. Section 4 constructs the generalized test statistic for the RDT plan on the MTTF of the system in Section 3. Section 5 illustrates the execution of the method through a numerical example and investigates the properties of the test plan through simulation studies. Section 6 presents the final conclusions.

## Notation list

**Table:** 

• *M*	real system MTTF;
• *M*_*c*_	requirement of the customer;
• *M*_*m*_	design level of the producer;
• *β*_*c*_	consumer’s risk;
• *β*_*m*_	manufacturer’s risk;
• *P*_*θ*_	the probability function under parameter vector *θ*;
• *T*_*s*_	system lifetime;
• *T*_*ij*_	the (latent) lifetime of *j*th component between *i*th and (*i* + 1)th component failure;
• *T*_*i*_	time between *i*th and (*i* + 1)th component failure;
• *T*(*X*;*x*,*ξ*)	generalized test statistic;
• $T_{e}(\bar{T};\bar{t},\xi )$	generalized test statistic for exponential case;
• $T_{w}(\bar{X,}S;\bar{x},s,\xi )$	generalized test statistic for Weibull distribution case;
• *W*_*c*_, *W*_*m*_, $W_{c}^{\prime }$, $W_{m}^{\prime }$	corresponding subsets of sample space.

## Materials and methods

### Problem formulations and methods

An RDT problem can be formulated from the perspective of statistical hypothesis testing with restrictions on the associated risks. When the risk-based restrictions are not strictly satisfied, the bias between the nominal and actual risks are considered as an optimization target function. Sometimes the system reliability function is replaced by other important reliability indices. Mean time to failure (MTTF) is an important product performance criterion from the perspective of reliability. It acts as a reference while determining maintenance and warranty policies. In this paper, we conduct a demonstration focusing on MTTF rather than system reliability at a given mission time. The problem can be formulated with the notations explained in the notation list as,

$$H_{0}:M\geq M_{c}↔H_{1}:\;M\leq M_{c}$$

$$satisfying\;risk\;restrictions\;:\;{sup}_{M\leq M_{c}}\;P_{\theta }(accept\;H_{0})\leq \beta_{c},{sup}_{M>M_{m}}\;P_{\theta }(refuse\;H_{0})\leq \beta_{m}$$

Sometimes it is not trivial to execute a hypothesis test on system MTTF. We denote the sample data of the test by *T* and the observed value by *t*. Conventional MLE based method can introduced for the test such as the test method for Weibull distribution in [28] and likelihood based method introduced in [29]. However, the approaches are all large sample based test plans which may cause significant bias when sample size is small. Further, since the method is always utilized for a single distribution, testing a function of multiple parameter will bring in great difficulties and further deviation. Noting that a conventional small sample based fixed-level test statistic for this problem is not available either, here we introduce a generalized test statistic based on the method introduced in [25]. For a random variable *X* having a pdf of *f*(*X*|*ξ*), where *ξ* = (*θ*,*η*) is a vector of unknown parameters. *θ* is the parameter of interest and *η* is the vector of nuisance parameters. A generalized test statistic of the form *T*(*X*;*x*,*ξ*) for a hypothesis test problem as defined in [25], is a function of not only *X* but also the observation *x* of *X* and parameter *ξ*.

**Definition 1.** We call *T*(*X*;*x*,*ξ*) a geralized test statistic if *T*(*X*;*x*,*ξ*) satisfis the following three requirements:

1. *T*(*x*;*x*,*ξ*) is free of *ξ*.
2. For fixed *x* and *ξ*, the distribution of *T*(*X*;*x*,*ξ*) is free of the nuisance parameter *η*.
3. For fixed *x* and *η*, the distribution function of *T*(*X*;*x*,*ξ*) nondecreasing in *θ*.

The definition of a generalized test statistic is a natural extension of the conventional test statistic that parameters and observed data are not involved. Condition a) is always considered applicable to the redundant cause and one just sets *T*(*X*;*x*,*ξ*) = *T*(*X*;*x*,*ξ*)−*T*(*x*;*x*,*ξ*) if condition a) does not hold. In this context, we can just suppose *T*(*x*;*x*,*ξ*) = 0 without loss of generality. Condition b) in the definition makes sure that parameters other than the parameter of interest have little influence on the distribution of the test statistic. Condition c) holds with respect to controlling the test power and risks. In our problem, the parameter vectors are multi-dimensional in both cases, while the parameter of particular interest (system MTTF) is unidimensional. By a simple transformation of the parameter space, a clear identification of *θ* and *η* can be made and generalized test methods can be implemented to establish a test plan. Once we establish a generalized test statistic satisfying the three principles above, it is natural to propose a test based on the p-value of this test statistic under an observed data *x*. Noticing that in the traditional p-value test plan for (1), an extreme region based on a conventional test statistic *T*(*X*) can be represented as

$$C_{x}(X)=(X:T(X)-T(x)>0)$$

where *x* is a pre-specified threshold for samples according to test level which satisfies the restriction. Let *ξ* = (*M*,*η*) where *η* is the vector of all nuisance parameters. Similar to the more traditional one-sided p-value test, the extreme region of a generalized test can be expressed in the following form

$$C_{x}(X,\xi )=\{X:T(X;x,\xi )-T(x;x,\xi )>0\}$$

Let the sample space be denoted by X. Considering the restrictions on both risks, we plan to establish a rejection region W⊂X satisfying

$$\left\{ \begin{array}{l} P_{M}\{x\notin W\}\leq \beta_{c}\\ P_{M}\{x\in W\}\leq \beta_{m} \end{array}\right.$$

We will describe the proposed way of establishing *W* in the following sections. Under some conditions and approximations, both the risk restrictions will be satisfied. Also numerical results from simulations will be presented.

## Load-sharing systems with exponential and Weibull components

In this section, we briefly review load-sharing systems subject to hard components failures, i.e., components are assumed to face sudden failures arising from traumatic shocks ([24]). As mentioned in the previous section, components in a load-sharing system are assumed to fail one by one. The whole system can be compiled as a parallel system model. The difference between a common parallel system and a load-sharing system lies in the variation of the working conditions of remaining components after one component fails. The lifetime distributions of components in different stages between adjacent component failures share different model parameters because of the redistribution of the total load among the remaining components; this is referred to as the load-sharing rule. Meanwhile, distribution of the family of lifetimes of the components is supposed not to vary in a given load-sharing system throughout its service life.

In a test for a load-sharing system, a number of *N* system samples in total are input into a lifetime test. Let us suppose the system consists of *I* components in total. During the test, the failure time of each component is observed until the total system fails. Let the time between (*i*-1)th and *i*th failures be denoted by *T*_*i*_ and the latent lifetime of each component when exact *i* components have failed in the system by *T*_*ij*_. The pass failed components’ data of each *T*_*ij*_ (not including the components that actually fail in each stage) can be considered as censored, which leads to being denoted by “latent” in our work. The parameter estimation still relies on the complete information including the data of those pass failed components and algorithms such as EM algorithm may be involved, but we don’t focus on this part in our work and related work on this can be found in [15] and [17]. For a fixed *i*, we assume a parameter vector, *θ*_*i*_, for all the distributions of *T*_*ij*_. The observed value of *T*_*i*_ in *n*th sample is the denoted by $t_{i}^{\left(n\right)}$, *n* = 1,⋯,*N*, then the data from the test is

$$t={\left(t^{\left(1\right)},\cdots ,t^{\left(N\right)}\right)}^{\prime }$$

where $t^{\left(n\right)}=\left(t_{1}^{\left(n\right)},\cdots ,t_{I}^{\left(n\right)}\right)$ is the observed data of *n*-th sample. Since *T*_*i*_ is dominated by the minimum lifetime among *T*_*ij*_, *i* = 1,⋯,*I*, *j* = 1,⋯,*I* + 1 − *i*, we have,

$$T_{i}=min\{T_{i1},\cdots ,T_{i(I-i+1)}\}$$

We will illustrate that under the assumption of an exponential or Weibull distribution, *T*_*i*_ is subject to the same family of distribution as *T*_*ij*_ due to the minimum closure property of exponential and Weibull distributions. Let the lifetime of the total system be denoted by *T*_*s*_, subject to the following relation obviously holds

$$T_{s}=\sum_{i=1}^{I}T_{i}$$

The MTTF of the whole system can be deduced from the following equation

$$M=E(T_{s})=E\left(\sum_{i=1}^{I}T_{i}\right)=\sum_{i=1}^{I}E(T_{i})=\sum_{i=1}^{I}\mu_{i}$$

where *μ*_*i*_ represents the mean of *T*_*i*_. In the following parts, we deduce the MTTF of the whole system based on these results. In a wide range of *load-sharing* systems, components are of similar types. Because of this, we make an assumption throughout the following parts of our work that the sets of model parameters for the remaining components in each stage between successive failures are independent from the components previously fail.

### Load-sharing systems with exponential components

Under the assumption of exponential distribution for each component, the underlying cumulative distribution function (CDF) of *T*_*ij*_ is supposed to be exponential:

$$F_{T_{ij}}(t)=1-exp\{-\lambda_{ij}t\}$$

where the failure rate of *j*th component once exactly (*i*-1) components have failed in the system is denoted by *λ*_*ij*_, due to the specific *load-sharing* rule, these *λ*_*ij*_ may not be equal. As we discussed above, *T*_*i*_ is the minimum of *T*_*i*1_,⋯,*T*_*i*(*I*−*i*+1)_. This may lead us to the distribution of *T*_*i*_ which can be written as

$$\begin{array}{l} F_{T_{i}}(t)=1-\prod_{j=1}^{I-i+1}P(T_{ij}>t)\\ \;=1-exp\left\{-\sum_{j=1}^{I-i}\lambda_{ij}t\right\}\\ \;=1-exp\{-\lambda_{i}t\} \end{array}$$

where $\lambda_{i}=\sum_{j=1}^{I-i}\lambda_{ij}$. From the above distribution, *T*_*i*_ is also subject to an exponential distribution with the parameter of failure rate $\lambda_{i}=\sum_{j=1}^{I-i}\lambda_{ij}$. This is a result of the minimum closure property of exponential distribution. We will show in the following parts that the same property is also shared by the more general case of Weibull distribution. Then MTTF of the system *M* under exponential distribution is

$$M=E(T_{s})=\sum_{i=1}^{I}\frac{1}{\lambda_{i}}$$

### Load-sharing systems with Weibull components

Now we consider the more general case of Weibull distribution. Under the assumption of a Weibull underlying distribution, the CDF of each *T*_*ij*_ is supposed to follow a Weibull distribution

$$F_{T_{ij}}(t)=1-exp\left\{-{\left(\frac{t}{\eta_{ij}}\right)}^{m_{i}}\right\}$$

where *η*_*ij*_ is the scale parameter of the CDF, and *m*_*i*_ is the shape parameter of the distribution of all *T*_*ij*_, *j* = 1,⋯,*I* + 1 − *i*. The shape parameter of a Weibull distribution is always supposed to characterize the distribution curve from the perspective of some common performance of similar products. Recalling that *T*_*ij*_ is the lifetime of each remaining component assuming that *i* components have failed, we can suppose that the distributions of all *T*_*ij*_ share the same shape parameter. Same as the discussion above, the distribution of *T*_*i*_ can be expressed as

$$F_{T_{i}}(t)=1-\prod_{j=1}^{I-i+1}exp\{-{\left(\frac{t}{\eta_{ij}}\right)}^{m_{i}}\}=1-exp\left\{-{\left(\frac{t}{\eta_{i}}\right)}^{m_{i}}\right\}$$

where $\eta_{i}={\left(\sum_{j=1}^{I-i+1}\frac{1}{{\eta_{ij}}^{m_{i}}}\right)}^{-\frac{1}{m_{i}}}$. From the distribution above, *T*_*i*_ is subject to a Weibull distribution with shape parameter *m*_*i*_ and scale parameter *η*_*i*_. Noting the mean of the Weibull distribution,

$$E(T_{i})=\eta_{i}∙\Gamma \left(1+\frac{1}{m_{i}}\right)$$

the MTTF of the system *M* can then be represented as

$$M=E(T_{s})=\sum_{i=1}^{I}\eta_{i}∙\Gamma (1+\frac{1}{m_{i}})$$

We have deduced the MTTF of the total system under an exponential or Weibull distribution so far. In the next section, we will propose test plans for demonstration executed on the performance of system MTTF.

## Test plan for load-sharing systems with exponential and Weibull components

In this section, we propose the detailed demonstration test plans under exponential and Weibull cases. We first start with the exponential case. Actually, the exponential distribution is a special case of Weibull distribution by pre-specifying the shape parameter. So the test plan for Weibull distribution can be naturally implemented in the exponential case with some modifications. Nevertheless, exponential distribution is a widely used lifetime model in practice. Meanwhile, the test plan for exponential case involves no approximations of model parameters which is necessary in the Weibull case. So we still spare a section for the exponential case first. In this section for exponential case, we also introduce an important property of fiducial inference which plays a critical role in the test plans for both cases.

### System RDT plan for load-sharing systems with exponential components

For a load-sharing system with exponential components, we let

$$\xi =(M,\eta )=(M,\lambda_{1},\cdots ,\lambda_{I})$$

where *η* = (*λ*_1_,⋯,*λ*_*I*_) is the vector of nuisance parameters. In a test for load-sharing systems, the observed value of *T*_*i*_ is recorded as the test data. Let ${\bar{t}}_{i}=\frac{1}{n}\sum_{n=1}^{N}t_{i}^{\left(n\right)}$, denote the observed data vector by $\bar{t}=({\bar{t}}_{1},\cdots ,{\bar{t}}_{I})$. For the convenience of the following part, we also need to denote a random variable ${\bar{T}}_{i}$ which has the same distribution as ${\bar{t}}_{i}$, and let $\bar{T}=({\bar{T}}_{1},\cdots ,{\bar{T}}_{I})$. Hence, the following pivot relation holds for each *λ*_*i*_,

$$2n\lambda_{i}{\bar{T}}_{i}∼\chi_{2n}^{2}$$

where $\chi_{2n}^{2}$ is the chi-square distribution with 2*n* degrees of freedom. We establish the generalized test statistic for *M* as

$$T_{e}(\bar{T};\bar{t},\xi )=\sum_{i=1}^{I}\frac{2n{\bar{t}}_{i}}{2n\lambda_{1}{\bar{T}}_{i}}-M=\sum_{i=1}^{I}\frac{{\bar{t}}_{i}}{\lambda_{1}{\bar{T}}_{i}}-M$$

From the definition of *T*_*e*_, it is easy to verify the following 3 relations:

1. Substitute $\bar{T}$ with the observed value ${\bar{t}}_{i}$, we have $T_{e}(\bar{t};\bar{t},\xi )$ = 0;
2. $T_{e}(\bar{T};\bar{t},\xi )\overset{d}{∼}\sum_{i=1}^{I}\frac{2n{\bar{t}}_{i}}{U_{i}}-M$, where $U_{1},\cdots ,U_{I}\overset{i.i.d}{∼}\chi_{2n}^{2}$ are independent Chi-square random variables. This means the distribution of *T*_*e*_ is free of nuisance parameter vector *η*.
3. The distribution of $T_{e}(\bar{T};\bar{t},\xi )$ is non-decreasing in *M*.

Hence, the test statistic $T_{e}(\bar{T};\bar{t},\xi )$ we define is a generalized test statistic according to [25]. To establish the rejection region, we first define two subsets of the sample space:

$$\left\{ \begin{array}{c} W_{c}=\{\bar{t}:P\{\sum_{i=1}^{I}\frac{2n{\bar{t}}_{i}}{U_{i}}\leq M_{c}\}\leq \beta_{c}\}\\ W_{m}=\{\bar{t}:P\{\sum_{i=1}^{I}\frac{2n{\bar{t}}_{i}}{U_{i}}\geq M_{m}\}\leq \beta_{m}\} \end{array}\right.$$

Let $\tilde{X}=W_{c}\cup W_{m}$ be the union of *W*_*c*_ and *W*_*m*_. Let *W* be any subset of the sample space and denote $\tilde{W}=W\cap \tilde{X}$ and ${\tilde{W}}^{c}=W^{c}\cap \tilde{X}$, where *W*^*c*^ represents the complement of *W*. We will show through series of interpretation that if *W* satisfies

$$\tilde{W}\subseteq W_{m},{\tilde{W}}^{c}\subseteq W_{c}$$

then *W* can be chosen as a rejection region which approximately satisfies the risk restrictions:

$$\left\{ \begin{array}{c} {sup}_{M\leq M_{c}}\;P_{M}\{\bar{t}\in W^{c}\}\leq \beta_{c}\\ {sup}_{M>M_{m}}\;P_{M}\{\bar{t}\in W\}\leq \beta_{M} \end{array}\right.$$

Before proceeding to further discussion, we need to make a statement without strict verification. We denote the cumulative distribution function of $\sum_{i=1}^{I}\frac{2n{\bar{t}}_{i}}{U_{i}}$ under fixed $\bar{t}$ by *F*_*U*_ where *U* = (*U*_1_,⋯,*U*_*I*_). For any confidence level 0 < *α* < 1, the following relation holds for any *M*

$$P_{M}\{\bar{t}:F_{U}^{-1}(1-\alpha )<M\}\approx \alpha$$

In fiducial inference, by replacing each $\frac{1}{\lambda_{i}}$ in *M* by $\frac{2n{\bar{t}}_{i}}{U_{i}}$, we have the pivot based fiducial distribution for *M* as $M\overset{d}{∼}\sum_{i=1}^{I}\frac{2n{\bar{t}}_{i}}{U_{i}}$. Actually, [30] pointed out that when *I* = 1, “≈” can be replace by “=” in the above equation which is an important frequency property of fiducial distribution based on pivots. Though theoretical work supporting this property when *I* > 1 has not been investigated, this phenomenon has already been noticed in literature several times and considered a fine property of pivot methods, which makes $F_{U}^{-1}(1-\alpha )$ a superior lower bound estimation for functions of parameters with confidence level *α*. Similar generalized fiducial method was also investigated for Weibull distribution in [31]. We will illustrate the numerical properties through simulations in following sections.

### System RDT plan for load-sharing systems with Weibull components

**Fig 1:**
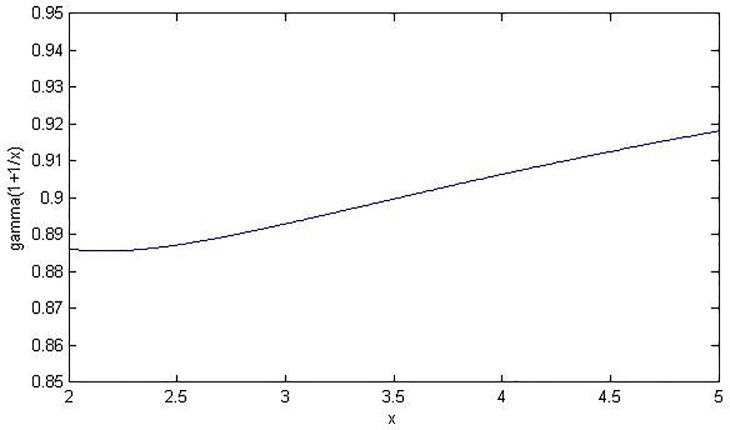
Sensitivity of the function $\Gamma \left(1+\frac{1}{X}\right)$ against *X* on the interval of (2, 5).

As for load-sharing systems with Weibull components, the MTTF of the system is represented in Eq (11). The item of each $\Gamma \left(1+\frac{1}{m_{i}}\right)$ increases the complexity of demonstration, however, the value of $\Gamma \left(1+\frac{1}{m_{i}}\right)$ is not sensitive to *m*_*i*_. The real shape parameter of a Weibull distribution is always considered to be within the rage of 2~5. In Fig 1, the value of $\Gamma \left(1+\frac{1}{m_{i}}\right)$ is shown against *m*_*i*_. The variation of $\Gamma \left(1+\frac{1}{m_{i}}\right)$ is quite insignificant from the figure and the relative deviation $\left(\Gamma (1+\frac{1}{2})-\Gamma (1+\frac{1}{5})\right)\cdot {\left(\Gamma (1+\frac{1}{2})\right)}^{-1}$ = 0.036. So, instead of presenting a demonstration based on the original *M*, we execute a demonstration on the “partial estimation” of system MTTF $\hat{M}=\sum_{i=1}^{I}\eta_{i}∙\Gamma (1+\frac{1}{{\hat{m}}_{i}})$ based on the estimation ${\hat{m}}_{i}$ of shape parameter *m*_*i*_. The numerical process for determining ${\hat{m}}_{i}$ can be found in [32].

Same as in the exponential case, we let

$$\xi =(\hat{M},\eta )=(\hat{M},\eta_{1},\cdots ,\eta_{I})$$

Let the log-transform of *T*_*i*_ be denoted by *X*_*i*_ = log*T*_*i*_, and the observed value of *X*_*i*_ in sample *n* by $x_{i}^{\left(n\right)}=\log t_{i}^{\left(n\right)}$. Let ${\bar{x}}_{i}=\frac{1}{N}\sum_{n=1}^{N}x_{i}^{\left(n\right)}$, $s_{i}^{2}=\frac{1}{N}\sum_{n=1}^{N}{\left(x_{i}^{\left(n\right)}-{\bar{x}}_{i}\right)}^{2}$. Let ${\bar{X}}_{i}$ and *S*_*i*_ respectively represent two statistics with the same distribution as ${\bar{x}}_{i}$ and *s*_*i*_. We have a pivot for each *η*_*i*_

$$\frac{{\bar{X}}_{i}-\ln\eta_{i}}{S}∼\frac{\bar{W}}{V}$$

where $\bar{W}=\frac{1}{N}\sum_{n=1}^{N}W_{i}$ and $V^{2}=\frac{1}{N}\sum_{n=1}^{N}{\left(W_{i}-\bar{W}\right)}^{2}$, $W_{1},\cdots ,W_{n}\overset{i.i.d.}{∼}1-e^{-e^{x}}$ is *n* independent random value with standard extreme value distribution. Let $\bar{X}=({\bar{X}}_{1},\cdots ,{\bar{X}}_{I})$, *S* = (*S*_1_,⋯,*S*_*I*_) and the observed data of $\bar{X}$ and *S* by $\bar{x}$ and *s* correspondingly. From this pivot, we can establish a generalized test statistic for $\hat{M}$ as

$$T_{w}(\bar{X,}S;\bar{x},s,\xi )=\sum_{i=1}^{I}\Gamma (1+\frac{1}{{\hat{m}}_{i}})\exp\left\{{\bar{x}}_{i}-\frac{{\bar{X}}_{i}-\ln\eta_{i}}{S}s_{i}\right\}-\hat{M}$$

denoting $\frac{{\bar{X}}_{i}-\ln\eta_{i}}{S}$ by *Z*_*i*_, we have $Z_{1},\cdots ,Z_{I}\overset{i.i.d.}{∼}\frac{\bar{W}}{V}$, and it is easy to verify that $T_{w}(\bar{X,}S;\bar{x},s,\xi )$ is a generalized test statistic. Also by defining the following two sets, $\left\{ \begin{array}{l} \;W_{c}^{\prime }=\{\bar{t}:P\{\sum_{i=1}^{I}\Gamma (1+\frac{1}{{\hat{m}}_{i}})\exp\left\{{\bar{x}}_{i}-Z_{i}s_{i}\right\}\leq M_{c}\}\leq \beta_{c}\}\\ W_{m}^{\prime }=\{\bar{t}:P\{\sum_{i=1}^{I}\Gamma (1+\frac{1}{{\hat{m}}_{i}})\exp\left\{{\bar{x}}_{i}-Z_{i}s_{i}\right\}\geq M_{m}\}\leq \beta_{m}\} \end{array}\right.$ in the same way as the exponential case, we can establish the rejection region *W*′ satisfying

$${\tilde{W}}^{\prime }\subseteq W_{m}^{\prime },{{\tilde{W}}^{\prime }}^{c}\subseteq W_{c}^{\prime }$$

where ${\tilde{W}}^{\prime }=W^{\prime }\cap \{W_{c}\cup W_{m}\}$, ${{\tilde{W}}^{\prime }}^{c}={W^{\prime }}^{c}\cap \{W_{c}\cup W_{m}\}$.

### Verification of the test plans for exponential and Weibull cases

In this section, we will verify that the generalized test statistics in the test plans under both cases satisfy the risk restrictions. We first verify that the risk restrictions holds if the rejection region *W* satisfying our restriction. Noting ${\tilde{W}}^{c}\subseteq W_{c}$ in (16), the following relation holds,

$$\begin{array}{l} sup_{M\leq M_{c}}\;P_{M}\left\{\bar{t}\in {\tilde{W}}^{c}\right\}\leq sup_{M\leq M_{c}}\;P_{M}\left\{\bar{t}\in W_{c}\right\}\\ \;=sup_{M\leq M_{c}}\;P_{M}\left\{\bar{t}:\;P\left\{\sum_{i=1}^{I}\frac{2n{\bar{t}}_{i}}{U_{i}}\leq M_{c}\right\}\leq \beta_{c}\right\}\\ \;=P_{M_{c}}\left\{\bar{t}:\;P\left\{\sum_{i=1}^{I}\frac{2n{\bar{t}}_{i}}{U_{i}}\leq M_{c}\right\}\leq \beta_{c}\right\}\\ \;=P_{M_{c}}\left\{\bar{t}:\;F_{U}^{-1}\left(\beta_{c}\right)\geq M_{c}\right\}\\ \;=1-P_{M_{c}}\left\{\bar{t}:\;F_{U}^{-1}\left(\beta_{c}\right)<M_{c}\right\}\\ \;\approx 1-\left(1-\beta_{c}\right)=\beta_{c} \end{array}$$

So ${sup}_{M\leq M_{c}}\;P_{M}\{\bar{t}\in {\tilde{W}}^{c}\}\leq \beta_{c}$.

Meanwhile, $\tilde{W}\subseteq W_{m}$ implies that

$$\begin{array}{l} {sup}_{M\geq M_{m}}\;P_{M}\{\bar{t}\in \tilde{W}\}\leq {sup}_{M\geq M_{m}}\;P_{M}\{\bar{t}\in W_{m}\}\\ \;=P_{M_{m}}\left\{\bar{t}:P_{M_{m}}\{\sum_{i=1}^{I}\frac{2n{\bar{t}}_{i}}{U_{i}}\geq M_{m}\}\leq \beta_{m}\right\}\\ \;=P_{M_{m}}\{\bar{t}:F_{U}^{-1}(1-\beta_{m})\leq M_{m}\}\\ \;\approx \beta_{m} \end{array}$$

So ${sup}_{M>M_{m}}\;P_{M}\{\bar{t}\in \tilde{W}\}\leq \beta_{m}$ holds.

Through the above deduction, we show that, with regard to the subset $\tilde{X}$, both restrictions on risks can be satisfied under our approach. The next step is to show that the probability of ${\tilde{X}}^{c}$ can be controlled. To verify this, we represent subset ${\tilde{X}}^{c}$ as

$$\begin{array}{l} {\tilde{X}}^{c}=\left\{\bar{t}:P_{M_{c}}\{\sum_{i=1}^{I}\frac{2n{\bar{t}}_{i}}{U_{i}}\leq M_{c}\}>\beta_{c},P_{M_{m}}\{\sum_{i=1}^{I}\frac{2n{\bar{t}}_{i}}{U_{i}}\geq M_{m}\}>\beta_{m}\right\}\\ \;=\{\bar{t}:F_{U}^{-1}(\beta_{c})<M_{c},F_{U}^{-1}(1-\beta_{m})\geq M_{m}\}\\ \;\subset \left\{\bar{t}:\frac{F_{U}^{-1}(\beta_{c})}{F_{U}^{-1}(1-\beta_{m})}<\frac{M_{c}}{M_{m}}\right\} \end{array}$$

meanwhile, when *n* → ∞, $\frac{2n}{U_{i}}\overset{p}{\to }1$ and ${\bar{t}}_{i}\overset{p}{\to }\frac{1}{\lambda_{i}}$, where “$\overset{p}{\to }$” represents convergence in probability. So the following relation holds,

$$\sum_{i=1}^{I}\frac{2n{\bar{t}}_{i}}{U_{i}}\overset{p}{\to }\sum_{i=1}^{I}\frac{1}{\lambda_{i}}=M$$

this means that, for any *β*_*c*_ and *β*_*m*_, $\frac{F_{U}^{-1}(\beta_{c})}{F_{U}^{-1}(\beta_{m})}\overset{p}{\to }1$. Noticing *M*_*c*_ < *M*_*m*_ implies $\frac{M_{c}}{M_{m}}<1$, then for any *ε* > 0, there exists an *n*_*ε*_, when *n* > *n*_*ε*_, for any *M*,

$$P_{M}\;\left\{\bar{t}:\frac{F_{U}^{-1}(\beta_{c})}{F_{U}^{-1}(\beta_{m})}<\frac{M_{c}}{M_{m}}\right\}<\varepsilon$$

specifically, this implies $P_{M}\{\bar{t}\in W\cap W_{1}^{c}\}\to 0$ as *n* → ∞. This means when *n* > *n*_*ε*_

$$\begin{array}{l} {sup}_{M\leq M_{c}}\;P_{M}\{\bar{t}\in W^{c}\}={sup}_{M\leq M_{c}}\;\left(P_{M}\{\bar{t}\in W^{c}\cap \tilde{X}\}+P_{M}\{\bar{t}\in W^{c}\cap {\tilde{X}}^{c}\}\right)\\ \;\leq {sup}_{M\leq M_{c}}P_{M}\{\bar{t}\in W^{c}\cap \tilde{X}\}+{sup}_{M\leq M_{c}}P_{M}\{\bar{t}\in {\tilde{X}}^{c}\}\\ \;\leq \beta_{c}+\varepsilon \end{array}$$

Then the second item $P_{M}\{\bar{t}\in W^{c}\cap {\tilde{X}}^{c}\}$, which introduces bias of test risks, can be further controlled under greater sample size *n*. The same conclusion can be drawn with regard to ${sup}_{M>M_{m}}\;P_{M}\{\bar{t}\in W\}$. We will illustrate the effectiveness of this approximation through simulations.

Following the same notation convention as the exponential case, let the distribution of $\sum_{i=1}^{I}\Gamma (1+\frac{1}{{\hat{m}}_{i}})\exp\left\{{\bar{x}}_{i}-Z_{i}s_{i}\right\}$ be denoted by *F*_*Z*_ where *Z* = (*Z*_1_,⋯,*Z*_*I*_). Since

$$P_{M}\{\bar{t}:F_{Z}^{-1}(1-\alpha )<M\}\approx \alpha$$

Similar to the exponential case, we can also conclude that the risk restrictions can be satisfied confined in the region of $W_{c}^{\prime }\cup W_{m}^{\prime }$, i.e., if *W*′ satisfies condition (23), then ${sup}_{M\leq M_{c}}\;P_{M}\{\bar{t}\in {{\tilde{W}}^{\prime }}^{c}\}\leq \beta_{c}$ and ${sup}_{M>M_{m}}\;P_{M}\{\bar{t}\in {\tilde{W}}^{\prime }\}\leq \beta_{m}$. Meanwhile, when *n* → ∞, by noting that fact that

$$\bar{W}\overset{p}{\to }\gamma ,\;V^{2}\overset{p}{\to }\frac{\pi^{2}}{6},{\bar{x}}_{i}\overset{p}{\to }\ln\eta_{i}+\frac{\gamma }{m_{i}},s_{i}^{2}\overset{p}{\to }\frac{\pi^{2}}{6m_{i}^{2}}$$

We have $\exp\left\{{\bar{x}}_{i}-\frac{{\bar{X}}_{i}-\ln\eta_{i}}{S}s_{i}\right\}\overset{p}{\to }\eta_{i}$ and $\sum_{i=1}^{I}\Gamma (1+\frac{1}{{\hat{m}}_{i}})\exp\left\{{\bar{x}}_{i}-\frac{{\bar{X}}_{i}-\ln\eta_{i}}{S}s_{i}\right\}\overset{p}{\to }\hat{M}$, moreover, as ${\hat{m}}_{i}\overset{p}{\to }m_{i}$, $\hat{M}\overset{p}{\to }M$. Similar conclusions can be drawn as the exponential case that

$$P_{M}\{\bar{t}\in {\left\{W_{c}\cup W_{m}\right\}}^{c}\}\to 0$$

The procedure of deduction is similar to those shown for the exponential case, they are omitted here.

## Realization of the test plan

In the previous section, we detailed the RDT plan based on a set of given data for the model. The realization of the test still depends on the calculation of $F_{U}^{-1}(a)$ and $F_{V}^{-1}(a)$ under a fixed level of *a*. The exact value of these two percentiles involves methods such as Mellin transform. The calculation process case unnecessary trouble, so we suggest sampling methods in our work. Generally, a wide range of simulation methods can be implemented here. As the scale (number of components) of the system increases, some sampling methods will suffer a significant complexity and estimation bias. In this section, we introduce a sampling method proposed in [33] for this problem. This method is not theoretically unbiased, however, it produces a fast and robust calculation. In the last of this section, we illustrate the execution of our method through a numerical example.

### Calculation of the test plan under exponential distribution

Let the *l*th percentile of *U*_*i*_ be denoted by *U*_*il*_, *l* = 1,⋯,*L*, where *L* is a preset large number. Given α ∈ (0,1), the confidence-level can be calculated through following steps:

***Step 1*.** Choose *M* pseudo random numbers *u*_1_,…,*u*_*M*_ from uniform distribution on (0, 1). Sort them in the order of value: *u*_(1)_ ≤ *u*_(2)_ ≤ ⋯ ≤ *u*_(*M*)_, we can rearrange any sequence {*U*_*il*_} as {*U*_*i*(*l*)_} according to the order of {*u*_(*l*)_}, repeat this process on {*U*_*il*_}, *i* = 1,⋯,*I* and denote the reordering result of each {*U*_*il*_} by $\left\{U_{i{\left(l\right)}^{i}}\right\}$. We have the following sampling result matrix below,
  $$\left[\begin{array}{ccc} U_{1{\left(1\right)}^{1}} & \cdots & U_{1{\left(L\right)}^{1}}\\ U_{2{\left(1\right)}^{2}} & \cdots & U_{2{\left(L\right)}^{2}}\\ \vdots & \ddots & \vdots \\ U_{I{\left(1\right)}^{I}} & \cdots & U_{I{\left(L\right)}^{I}} \end{array}\right]$$
  where (*l*)^1^,⋯,(*l*)^*I*^ denote the resampling subscripts.
***Step 2*.** Given a confidence level of *a*, Calculate:
  $$F_{l}=\sum_{i=1}^{I}\frac{2n{\bar{t}}_{i}}{U_{i{\left(l\right)}^{i}}},\;l=1,2,\cdots ,L$$
  Then sort {*F*_1_,⋯,*F*_*L*_} according to the values as the sampling results {*F*_(1)_,⋯,*F*_(*L*)_} and so the α-level right percentile of *T*(*Y*;*y*,*ξ*) can be chosen from these sampling results as *F*_⌊(1−*α*)*M⌋_.

### Calculation of the test plan under Weibull distribution

Since the two pivots $\bar{W}$ and *V* in the Weibull case are dependent statistics generated from standard extreme value distributions, we need to make some modifications to implement the procedure in exponential for the Weibull case.

***Step 1*.** Choose *w*_*i*_ as *i*-th percentiles of standard extreme value distribution, specifically, $w_{i}=\ln(-\ln\left(1-\frac{i}{K+1}\right)),i=1,\cdots L$, then execute the procedure of allocating order method on *w*_1_,⋯,*w*_*K*_ for *L* times, denote the sampling result as below:
  $$\left[\begin{array}{ccc} w_{{\left(1\right)}^{1}} & \cdots & w_{{\left(L\right)}^{1}}\\ \vdots & \ddots & \vdots \\ w_{{\left(1\right)}^{L}} & \cdots & w_{{\left(L\right)}^{L}} \end{array}\right]$$
***Step 2*.** Generate the sampling results of $\bar{W}$ and *V*^2^ using the results in ***Step 1*** and then generate the sampling result of *Z*_1_ in the following way:
  $$\left[\begin{array}{ccc} {\bar{w}}_{1}=\frac{1}{L}\sum_{i=1}^{L}w_{{\left(1\right)}^{i}} & , & V_{1}^{2}=\frac{1}{L}\sum_{i=1}^{L}{\left(w_{{\left(1\right)}^{i}}-{\bar{w}}_{1}\right)}^{2}\\ \vdots & & \vdots \\ {\bar{w}}_{L}=\frac{1}{L}\sum_{i=1}^{L}w_{{\left(L\right)}^{i}} & , & V_{L}^{2}=\frac{1}{L}\sum_{i=1}^{L}{\left(w_{{\left(L\right)}^{i}}-{\bar{w}}_{L}\right)}^{2} \end{array}\right]\to \left[\begin{array}{c} Z_{1{\left(1\right)}^{1}}=\frac{{\bar{w}}_{1}}{V_{1}}\\ \vdots \\ Z_{1{\left(L\right)}^{1}}=\frac{{\bar{w}}_{L}}{V_{L}} \end{array}\right]$$
***Step 3*.** Repeat the previous two steps for all *Z*_1_∼*Z*_*I*_, we have the sampling results:
  $$\left[\begin{array}{ccccc} Z_{1{\left(1\right)}^{1}} & , & \cdots & , & Z_{I{\left(1\right)}^{I}}\\ \vdots & & & & \vdots \\ Z_{1{\left(L\right)}^{1}} & , & \cdots & , & Z_{I{\left(L\right)}^{I}} \end{array}\right]$$
***Step 4*.** Given a confidence level, *a*, calculate
  $$F_{l}=\sum_{i=1}^{I}\exp\left\{{\bar{x}}_{i}-Z_{i{\left(1\right)}^{i}}s_{i}\right\}$$

Again, as in the exponential case, sort {*F*_1_,⋯,*F*_*L*_} according as {*F*_(1)_,⋯,*F*_(*L*)_}, so the α-level right percentile of *T*(*Y*;*y*,*ξ*) can be chosen from these sampling results as *F*_⌊(1−*α*)*M⌋_.

### A numerical example

We now illustrate the execution of our method through a numerical example. Both risks are set at 0.1. The number of components *I* is set at 3 and the sample size *n* in the test is set at 10. In the exponential case, the threshold from customer and manufacturer are fixed at *M*_*m*_ = 1.5 and *M*_*c*_ = 1.2 correspondingly and failure rates of each stage are pre-specified in Table 1.

**Table 1:** Pre-specified parameters for exponential numerical example.

Stage	Component 1 failure rate	Component 2 failure rate	Component 3 failure rate	System failure rate
1	*λ*_11_ = 0.2	*λ*_12_ = 0.3	*λ*_13_ = 0.5	*λ*_1_ = 1.0
2	NA	*λ*_21_ = 0.5	*λ*_22_ = 1.5	*λ*_2_ = 2.0
3	NA	NA	*λ*_31_ = 3.0	*λ*_3_ = 3.0

In each stage, we use the pre-specified parameters to simulate the lifetimes of all remaining component. Then we choose the component with the minimum lifetime among them as the actually failed component. We denote the lifetime time of this component as the underlying observed failure time. We repeat the same simulation procedure for all 10 samples. The simulated data are shown in Table 2.

**Table 2:** Simulated data set for exponential numerical example.

Failure time series Sample No.	Component failure time 1	Component failure time 2	Component failure time 3
1	0.2049	0.1740	0.2746
2	0.0989	0.7236	0.4412
3	2.0637	0.5420	0.2891
4	0.0906	1.1377	0.0764
5	0.4583	1.0658	0.5591
6	2.3275	0.0971	0.3379
7	1.2383	0.1820	0.2695
8	0.6035	0.5743	0.2455
9	0.0434	0.0255	0.1145
10	0.0357	1.3842	0.0938

The sample mean for simulated data set is ${\bar{t}}_{1}=0.76$, ${\bar{t}}_{2}=0.59$, ${\bar{t}}_{3}=0.27$. The simulation method described in Section 4.1, yields the following percentiles: $\sum_{i=1}^{I}\frac{2n{\bar{t}}_{i}}{U_{i}}$ are $F_{U}^{-1}(0.1)=1.27$ and $F_{U}^{-1}(0.9)=1.76$. Since $F_{U}^{-1}(0.1)\geq 1.2$ and $F_{U}^{-1}(0.9)\geq 1.5$, it implies that (0.66,0.59,0.27) ∈ *W*_*c*_ and (0.66,0.59,0.27) ∉ *W*_*m*_, so we should reject null hypothesis.

In the Weibull case, we preset the two thresholds at *M*_*m*_ = 4 and *M*_*c*_ = 5, respectively. Next, we preset the parameters in Table 3.

**Table 3:** Pre-specified parameters for Weibull numerical example.

Stage	Component 1 scale parameter	Component 2 scale parameter	Component 3 scale parameter	System scale parameter	Shape parameter
1	*η*_11_ = 1.7	*η*_12_ = 1.8	*η*_13_ = 1.9	*η*_1_ = 1.03	*2*.*0*
2	NA	*η*_21_ = 1.2	*η*_22_ = 1.3	*η*_2_ = 0.88	*2*.*0*
3	NA	NA	*η*_31_ = 0.8	*η*_3_ = 0.80	*2*.*0*

We simulate the data in the same way as the exponential case. The simulated data is shown in Table 4.

**Table 4:** Simulated data set for Weibull numerical example.

Failure time series Sample No.	Component failure time 1	Component failure time 2	Component failure time 3
1	0.3527	0.5717	0.7261
2	0.3146	1.6064	0.7853
3	1.3365	0.3559	0.4135
4	0.4010	0.2300	0.3830
5	0.6770	0.5478	1.0361
6	1.4256	0.4635	0.6759
7	1.1306	0.4795	0.7193
8	0.7769	0.8513	0.5285
9	0.3084	0.4719	0.4688
10	0.3891	1.0691	0.4244

The sample mean and variance of simulated data are ${\bar{x}}_{1}=0.70$, *s*_1_ = 0.49, ${\bar{x}}_{2}=0.66$, *s*_2_ = 0.41, ${\bar{x}}_{3}=0.62$, *s*_3_ = 0.21. The estimation of *m*_*i*_’s are: ${\hat{m}}_{1}=2.13$, ${\hat{m}}_{2}=2.11$, ${\hat{m}}_{2}=2.07$. So the parameter to be demonstrated is

$$\hat{M}=\sum_{i=1}^{I}\eta_{i}∙\Gamma (1+\frac{1}{{\hat{m}}_{i}})=0.88\eta_{1}+0.88\eta_{2}+0.89\eta_{3}$$

Through the simulation procedure presented in section 4.2 for the Weibull case, the results of percentiles, $\sum_{i=1}^{I}\Gamma (1+\frac{1}{{\hat{m}}_{i}})\exp\left\{{\bar{x}}_{i}-Z_{i}s_{i}\right\}$, are recalculated as $F_{Z}^{-1}(0.9)=5.13>5$, so we should accept the null hypothesis.

## Simulation studies

### Verification of $F_{U}^{-1}$ and $F_{Z}^{-1}$

In this section, we investigate the properties of our method through simulation studies. Recall the test plans for both exponential case and Weibull case enable some flexibility for the rejection region. The restrictions on risks are on the basis of the premise of $P_{M}\{\bar{t}:F_{U}^{-1}(1-\alpha )<M\}\approx \alpha$ and $P_{M}\{\bar{t}:F_{Z}^{-1}(1-\alpha )<M\}\approx \alpha$. However, as stated in previous sections, except for the special case where *I* equals 1, the approximation of the real coverage rate of $F_{U}^{-1}$ and $F_{Z}^{-1}$ as estimated lower limits of real MTTF has not been verified. The bias of the real coverage from *α* may cause bias of actual test risks from nominal risks *β*_*c*_ and *β*_*m*_. First, we will illustrate the real coverage rate, i.e., $P_{M}\{\bar{t}:F_{U}^{-1}(1-\alpha )<M\}$ and $P_{M}\{\bar{t}:F_{Z}^{-1}(1-\alpha )<M\}$ through simulations. We simulate a series of data set under pre-specified model parameters, and observe the ratio of trials which makes the relation $F_{U}^{-1}(1-\alpha )<M$ and $F_{Z}^{-1}(1-\alpha )<M$ hold. The number of simulations in both exponential and Weibull cases are 1000 and sample size *n* = 20. The simulation results are listed in Table 5 and Table 6.

**Table 5:** Coverage rate of simulations results $F_{U}^{-1}(1-\alpha )$ as an estimated lower limit of system reliability subject to exponential component lifetime distribution.

**Actual system MTTF**	**Number of Components** ***I***	**Confidence level α = 0.20**	
		Coverage rate	1-α Percentile of simulated values
**15.62**	2	0.207	14.81
**18.31**	3	0.211	16.76
**22.44**	4	0.213	20.13
	Number of Components *I*	Confidence level α = 0.10	
		Coverage rate	1-α Percentile of simulated values
**15.62**	2	0.112	14.47
**18.31**	3	0.117	16.55
**22.44**	4	0.119	19.31

**Table 6:** Coverage rate of simulations results $F_{Z}^{-1}(1-\alpha )$ as an estimated lower limit of system reliability subject to Weibull component lifetime distribution.

**Actual system MTTF**	**Number of Components** ***I***	**Confidence level α = 0.20**	
		Coverage rate	1-α Percentile of simulated values
**33.27**	2	0.213	31.11
**40.36**	3	0.219	37.26
**50.68**	4	0.221	48.24
	Number of Components *I*	Confidence level α = 0.10	
		Coverage rate	1-α Percentile of simulated values
**33.27**	2	0.118	30.67
**40.36**	3	0.122	36.44
**50.68**	4	0.125	45.93

Results in Tables 5 and Table 6 reveal a uniform significant conservative actual coverage rate under both exponential and Weibull distributions. The relation “≈”may actually be “≥” in the case of the load-sharing systems in our work. Through a consistent notation *F*^−1^ for both $F_{U}^{-1}$ and $F_{Z}^{-1}$, this will cause bias of the actual manufacturer’s risk from *β*_*m*_ through the following relation: $P_{M_{m}}\{\bar{t}:\;F^{-1}(1-\beta_{m})\leq M_{m}\}\geq \beta_{m}$ To overcome this potential bias, recall that we can choose arbitrary *W* satisfying $\tilde{W}\subseteq W_{m}$ and ${\tilde{W}}^{c}\subseteq W_{c}$, so we can just choose $W=\tilde{W}=W_{m}\backslash \{W_{m}\cap W_{c}\}$ in the following equation

$$P_{M}\{\bar{t}\in \tilde{W}\}=P_{M}\{\bar{t}\in W_{m}\}-P_{M}\{\bar{t}\in W_{m}\cap W_{c}\}$$

The probability of *W*_*m*_ ∩ *W*_*c*_ will reduce the discrepancy between the actual risk and the nominal manufacturer’s risk *β*_*m*_ to some degree. In the next subsection, we will illustrate the actual risks under such a rejection region *W*.

### Actual test risks

In the previous sections, we establish test plans for load-sharing systems subject to exponential and Weibull components. The test plans are based on small sample generalized pivots with exact distributions. However, a series of approximations are made in the tests. Note that, inevitably, a vague region, $W^{c}\cap {\tilde{X}}^{c}$, appears in the sample space. These regions will cause biases in actual risks (in comparison with the nominal ones). We will now investigate actual risks of test plans under different conditions. We generate the simulated samples in the same way as the previous section of the numerical example. For simplicity, we just show the pre-specified system level parameters in each stage in this section.

In the case of exponential underlying distribution, we choose a system with *I* = 3 components in total. At each stage, we pre-specify the parameter of failure rate: *λ*_1_ = 2, *λ*_2_ = 3, *λ*_3_ = 4 and simulate a sample set of size *N* under corresponding failure rate. We execute the test plan with the simulated sample set and repeat the process for 1000 times. We calculate the actual test risks through this 1000 tests and the results are listed in Table 7.

**Table 7:** Actual test risks under exponential distribution.

Sample size	Nominal Consumer’s Risk	Nominal manufacturer’s risk	Actual consumer’s risk	Actual manufacturer’s risk
N	*β*_*c*_	*β*_*m*_	$\beta_{c}^{\prime }$	$\beta_{m}^{\prime }$
**10**	0.1	0.1	0.107	0.112
**10**	0.15	0.15	0.157	0.163
**15**	0.1	0.1	0.106	0.092
**15**	0.15	0.15	0.145	0.155
**20**	0.1	0.1	0.109	0.095
**20**	0.15	0.15	0.141	0.159

Table 7 compares the actual risks with the nominal ones. The results reveal a bias with respect to test risks. We can see that the bias can be controlled within the range of 2% under different conditions. However, the biases associated with the two types of risks cannot be controlled simultaneously. When the sample size is small, the bias shows more significance due to the region of $W^{c}\cap {\tilde{X}}^{c}$. However, the bias does not show much sensitivity to sample size.

In the case of Weibull underlying distribution, the number of components is also set at *I* = 3. The model parameters are pre-specified as: (*η*_1_,*m*_1_) = (1,2), (*η*_2_,*m*_2_) = (2,3), (*η*_3_,*m*_3_) = (3,4). The sample size is set at 3 levels: 10, 15 and 20. The results of actual risks through 1000 simulations for each sample size are illustrated in Table 8.

**Table 8:** Actual test risks under Weibull distribution.

Sample size	Nominal Consumer’s Risk	Nominal manufacturer’s risk	Actual consumer’s risk	Actual manufacturer’s risk
N	*β*_*c*_	*β*_*m*_	$\beta_{c}^{\prime }$	$\beta_{m}^{\prime }$
**10**	0.1	0.1	0.112	0.117
**10**	0.15	0.15	0.143	0.166
**15**	0.1	0.1	0.091	0.122
**15**	0.15	0.15	0.158	0.161
**20**	0.1	0.1	0.103	0.11
**20**	0.15	0.15	0.156	0.163

From Table 8, the biases between test risks and nominal ones are uniformly larger than the exponential case. Since we are calculating actual risks here, we use the real value of *m*_*i*_ for the target MTTF of the whole system. Note that the estimated value ${\hat{m}}_{i}$ of *m*_*i*_ introduces deviation of $\hat{M}$ around the real value *M*. As we establish our test statistic based on the former, the additional bias of test risks caused by this procedure are also presented in Table 8.

In both exponential and Weibull cases, we have determined the biases observed through tests. Like many other demonstration methods incorporating approximation techniques, the bias of test risks are significant compared to exact methods. However, for a multi-stage test problem such as a load-sharing demonstration, although a proper exact method cannot be established or even does not exist, approximation methods seems to show great strengths. Compared to some demonstration test plans in other famous standards such as MIL-HDBK-781A (1987) and IEC-1123 (1991), the biases are acceptable. Meanwhile, the method is not sensitive to sample size which makes it available when the sample size is not large enough for a large sample based method. The fast numerical solution procedure described in Section 4 endows the method more facilitative in practice.

## Concluding remarks

In this paper, we have studied a test scheme for RDT on load-sharing systems with exponential and Weibull component lifetime distributions. For such demonstration problems where multiple parameters are involved, conventional MLE based large sample test methods may introduce bias of risks. We introduce the generalized test statistics for this specific type of demonstration test. By considering all parameters other than the target index of system MTTF, we establish generalized test statistics for both exponential and Weibull cases. The corresponding rejection regions have been established according to the generalized test statistics. The problem caused by miscellaneous model parameters during demonstration test is effectively solved through this method.

The test plans introduce approximations and biases of test risks have been introduced because of these approximations. In view of this, these additional improvements can be further studied to reduce such biases. The generalized test statistic is an effective tool for hypothesis test problems with multiple parameters. The generalized test statistic can be developed on the basis of ordinary pivots for parameters of conventional parametric lifetime distributions, i.e., exponential, Weibull, normal, lognormal, binomial. When conventional pivots are unavailable for the models, the establishment of the generalized test statistics will meet more challenge. Meanwhile, to establish a generalized test statistic, an explicit and closed form of target index (system reliability, MTTF, failure rate etc) with respect to the parameters is necessary. This explicit function form is also accessible in some other important systems such as parallel systems and *k-out-of-n* systems by considering system reliability as demonstration target. These may all lead to other potential extensions of the implementation of the generalized statistics method in more general problems.
